# Exploring genetic associations between metabolites and atopic dermatitis: insights from bidirectional Mendelian randomization analysis in European population

**DOI:** 10.3389/fnut.2024.1451112

**Published:** 2024-09-10

**Authors:** Ao He, Zhisheng Hong, Xinqi Zhao, Hainan Li, Ying Xu, Yangheng Xu, Zhaoyi Jing, Haoteng Ma, Zhuo Gong, Bing Yang, Qingzhu Zhou, Fan Zheng, Xian Zhao

**Affiliations:** ^1^The Affiliated Calmette Hospital of Kunming Medical University, Plastic Surgery/Kunming First People’s Hospital, Plastic Surgery, Kunming, Yunnan, China; ^2^The Second School of Clinical Medicine, Southern Medical University, Guangzhou, Guangdong, China; ^3^The First School of Clinical Medicine, Southern Medical University, Guangzhou, Guangdong, China; ^4^Shandong University of Traditional Chinese Medicine, Jinan, Shandong, China

**Keywords:** atopic dermatitis, blood metabolites, genome-wide association study, Mendelian randomization, causal inference

## Abstract

**Introduction:**

There is growing evidence indicating a complex interaction between blood metabolites and atopic dermatitis (AD). The objective of this study was to investigate and quantify the potential influence of plasma metabolites on AD through Mendelian randomization (MR) analysis.

**Methods:**

Our procedures followed these steps: instrument variable selection, primary analysis, replication analysis, Meta-analysis of results, reverse MR analysis, and multivariate MR (MVMR) analysis. In our study, the exposure factors were derived from the Canadian Longitudinal Study on Aging (CLSA), encompassing 8,299 individuals of European descent and identifying 1,091 plasma metabolites and 309 metabolite ratios. In primary analysis, AD data, was sourced from the GWAS catalog (Accession ID: GCST90244787), comprising 60,653 cases and 804,329 controls. For replication, AD data from the Finnish R10 database included 15,208 cases and 367,046 controls. We primarily utilized the inverse variance weighting method to assess the causal relationship between blood metabolites and AD.

**Results:**

Our study identified significant causal relationships between nine genetically predicted blood metabolites and AD. Specifically, 1-palmitoyl-2-stearoyl-GPC (16:0/18:0) (OR = 0.92, 95% CI 0.89–0.94), 1-methylnicotinamide (OR = 0.93, 95% CI 0.89–0.98), linoleoyl-arachidonoyl-glycerol (18:2/20:4) [1] (OR = 0.94, 95% CI 0.92–0.96), and 1-arachidonoyl-GPC (20:4n6) (OR = 0.94, 95% CI 0.92–0.96) were associated with a reduced risk of AD. Conversely, phosphate / linoleoyl-arachidonoyl-glycerol (18:2/20:4) [2] (OR = 1.07, 95% CI 1.04–1.10), docosatrienoate (22:3n3) (OR = 1.07, 95% CI 1.04–1.10), retinol (Vitamin A) / linoleoyl-arachidonoyl-glycerol (18:2/20:4) [2] (OR = 1.08, 95% CI 1.05–1.11), retinol (Vitamin A) / linoleoyl-arachidonoylglycerol (18:2/20:4) [1] (OR = 1.08, 95% CI 1.05–1.12), and phosphate / linoleoyl-arachidonoyl-glycerol (18:2/20:4) [1] (OR = 1.09, 95% CI 1.07–1.12 were associated with an increased risk of AD. No evidence of reverse causality was found in the previously significant results. MVMR analysis further confirmed that 1-palmitoyl-2-stearoyl-GPC (16:0/18:0) and 1-methylnicotinamide are independent and dominant contributors to the development of AD.

**Conclusion:**

Our study revealed a causal relationship between genetically predicted blood metabolites and AD. This discovery offers specific targets for drug development in the treatment of AD patients and provides valuable insights for investigating the underlying mechanisms of AD in future research.

## 1 Introduction

Atopic dermatitis (AD) is a common chronic skin inflammatory disease that typically starts during childhood. Severe itching, which leads to scratching and further skin damage, affecting approximately 30% of people worldwide (1). The disease imposes a significant economic burden on patients, families, and society at large, resulting in both direct healthcare costs and decreased productivity. The cause of AD is intricate and involves a variety of factors, including genetic vulnerability. Mutations in the filament polyprotein gene (FLG) can increase epidermal water loss, increasing the vulnerability of the skin to irritants and pathogens. Despite extensive research on the molecular mechanisms and biological processes of AD, current measures of disease severity are flawed (2).

AD progression is significantly impacted by immune system effects, leading to decreased levels of carnitine, essential amino acids, and related compounds due to cytokine dysregulation. It is worth noting that although these results provide ample evidence of the potential of blood metabolites as biomarkers of AD, the conclusions derived from various studies are not completely uniform because of the inherent constraints of observational research.

Mendelian randomization (MR) leverages genetic variability from GWAS data as an instrumental variable to evaluate causality between exposures and outcomes, mimicking randomized controlled trials (3, 4). This method is used when traditional trials are impractical. Several previous MR analyses have investigated the causal relationships between certain metabolites and AD. Nevertheless, Previous MR studies on metabolites and AD have been limited in scope. Consequently, in this study, the most recent and comprehensive GWAS data on blood metabolites and AD were selected for extensive MR analysis. This study was performed with the aim of comprehensively elucidating the potential mechanisms underlying the causal relationship between blood metabolites and AD, which will provide a reliable basis for the formulation of feasible AD therapeutic and prophylactic approaches in clinical practice.

## 2 Materials and methods

### 2.1 Study design

**FIGURE 1:**
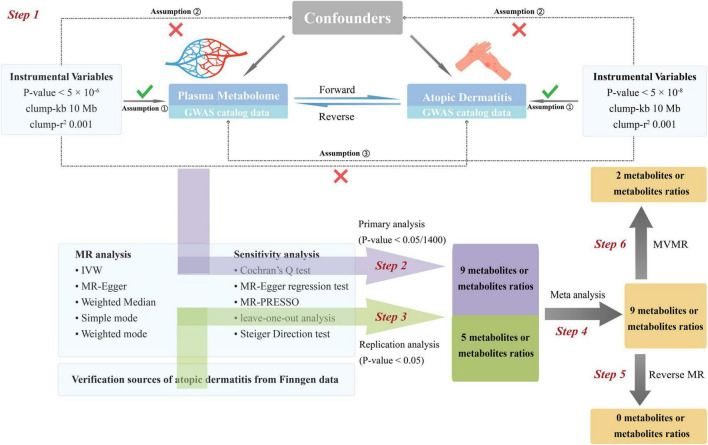
Flow chat of fundamental assumptions and analysis approaches in MR study design. Step 1: Instrument variable selection: We meticulously identified instrumental variables (IVs) that meet the three key Mendelian randomization (MR) assumptions and exhibit strong correlation with the exposure of interest. Step 2: Primary analysis: Utilizing a two-sample MR approach, we scrutinized the causal relationship between 1,400 metabolites and their ratios (as the exposure variables, with original data sourced from the GWAS catalog) and atopic dermatitis (AD) (as the outcome variable, also derived from the GWAS catalog). Multiple sensitivity analyses were conducted to ensure the robustness of our findings. By applying Bonferroni correction, the significance threshold was set at 0.05/1,400, leading to the identification of 9 significant metabolites and their ratios. Step 3: Replication analysis: We conducted a replication analysis using AD GWAS data from the FinnGen database, designated as the outcome variable. The significance threshold was set at 0.05. Applying the same MR analysis protocol as in the primary analysis to the 9 significant findings, we identified 5 metabolites and their ratios that remained significant. Step 4: Meta-analysis of results: A meta-analysis was performed on the results from both the primary and replication analyses, confirming the significance of 9 metabolites and their ratios. Step 5: Reverse Mendelian randomization analysis: In a bid to further validate our results, we performed a reverse MR analysis by swapping the exposure and outcome variables from the primary analysis—using AD data as the exposure and metabolites as the outcome. The findings indicated no evidence of reverse causation between metabolites and AD, thus enhancing the credibility of our conclusions. Step 6: Multivariate MR analysis: The 9 significant results from Step 4 were categorized into 5 individual metabolites and 4 metabolite ratios. Multivariate MR analysis revealed that 2 metabolites exert independent causal effects on AD, distinct from the influence of other metabolites. IVs, instrumental variables. MR, Mendelian randomization (MR). Kb, Kilobase pairs. r2, coefficient of determination. IVW, inverse variance weighting method. MR-PRESSO, Mendelian Randomization Pleiotropy RESidual Sum and Outlier.

The flowchart of this study is depicted in Figure 1. The most recent and comprehensive GWAS pooled datasets were utilized. IVs were screened based on the three major assumptions of MR (5). We performed forward MR analysis to infer causal relationships between blood metabolites and AD. The post-MR meta-analysis was added to enhance the dependability of the findings. Additionally, reverse MR analysis was employed to examine the causative relationship between AD and blood metabolites. Finally, multivariable Mendelian randomization (MVMR) was used to determine whether metabolites identified as having significant causal relationships with AD were independently associated with AD. The publicly available GWAS data used in this study rendered it unnecessary to procure informed consent or ethical approval. Additionally, we followed the MR guidelines from the Strengthening the Reporting of Observational Studies in Epidemiology (STROBE) (Supplementary Data Sheet 1) (6). No ethical approval was required for the present study, for all data sources were based on publicly available summary-level data.

### 2.2 Data sources

For exposure factors, our research is based on data from the Canadian Longitudinal Study on Aging (CLSA), a nationally representative sample from all ten Canadian provinces (7). The dataset includes a wide range of information and biospecimens to address various research inquiries on aging. The CLSA consists of around 51,338 Canadian adults of both genders (with the gender ratio approximating 1:1), aged between 45 and 85 at recruitment. It provides a diverse collection of biological, medical, physiological, social, lifestyle, and economic data, offering a comprehensive view of the aging process. Our focus is on 8,299 unrelated individuals of European descent within the CLSA, who have undergone whole-genome genotyping and plasma metabolite measurements. This allows for a detailed investigation into the genetic and metabolic aspects of aging-related conditions. We obtained summary statistics for publicly available blood metabolomics data from the GWAS catalog for exposure factors. Our thorough analysis identified 1,091 plasma metabolites and 309 metabolite ratios (8). The characteristics of the selected blood metabolite datasets are detailed in Supplementary Tables 1A, B.

We obtained AD summary statistics as a discovery cohort from the GWAS catalog (accession number GCST90244787). This is the most extensive genome-wide association study (GWAS) of AD conducted to date (9). This comprehensive study has successfully identified 91 robustly associated loci, with 22 exhibiting suggestive evidence of population-specific effects. Our analysis incorporated GWAS data across 40 cohorts, comprising 60,653 individuals with AD and 804,329 controls of European descent. Notably, the cohorts included a substantial pediatric component, with 25 cohorts focusing on children, alongside 15 adult cohorts. While the gender ratio remains undisclosed, the collective characteristics of the selected datasets for AD are delineated in Supplementary Table 1C, providing a transparent foundation for our study’s methodology and findings.

Furthermore, for the replication cohort, we employed summary data from individuals of European ancestry derived from the Finnish R10 database, encompassing 15,208 cases of AD and an extensive control group of 367,046 individuals. The FinnGen study, a monumental genomics initiative, has meticulously analyzed over 500,000 Finnish biobank samples, elucidating the correlation between genetic variations and health profiles to unravel the intricacies of disease mechanisms and predispositions. This collaborative endeavor encompasses Finnish research institutions, biobanks, and international industry partners, fostering a robust framework for genetic research. As of 18 December 2023, the FinnGen R10 cohort has amassed a total sample size of 412,181 participants, with a gender distribution of 230,310 females and 181,871 males. The median age of the participants, which stands at 63 years, coupled with a significant portion recruited from hospital settings, has notably enriched the disease endpoints within the FinnGen study, providing a unique and valuable perspective on the genetic underpinnings of AD in a mature demographic. The characteristics of the selected AD datasets are shown in Supplementary Table 1C.

### 2.3 The selection of instrumental variables

In this study, the independent variables (IVs) underwent thorough screening proposed by MR. Specifically, (1) for the AD data, the genome-wide significance threshold we employed was a *P*-value (*P*) < 5 × 10^–8^. For blood metabolite phenotypes, since the conventional threshold *P* < 5 × 10^–8^, a sufficient number of SNPS may not be screened to be included in subsequent analyses. *P* < 5 × 10^–6^ is used as a lenient screening criterion to secure an adequate number of IVs to ensure reliable results. (2) Data from the 1,000 Genomes Project Europe samples were employed by the reference panel to calculate linkage disequilibrium (LD) between single nucleotide polymorphisms (SNPs). IVs without linkage effects were included for further analysis (*r*^2^ < 0.001, window size = 10 Mb). (3) The strength of individual SNPs was tested by calculating the F-statistic using the formula [(N-K-1)/K] × [R2/(1-R2)], where R2 indicates the proportion of exposure variance explained by IVs. To mitigate weak instrumental variable bias, SNPs with F-statistics less than 10 were excluded from subsequent analysis. (4) Any mismatched palindromic SNPs were removed, while the SNPs were adjusted to always be associated with the same allele to ensure reliable study results.

### 2.4 Primary analysis

To assess the causal relationship between blood metabolites and AD, the inverse variance weighted (IVW) method, along with the MR-Egger method and the weighted median (WM) method, were used for bidirectional MR analysis. Where horizontal pleiotropy is absent, IVW offers the most precise estimations. Additional methods were employed to supplement the IVW findings, and the consistent direction of the effect size from various methods was considered indicative of stable and reliable results. The IVW method leverages a broad spectrum of genetic variations, yielding consistent and systematic outcomes. When the validity of genetic variants is compromised, with more than half being potentially invalid, the WM method becomes particularly crucial. In contrast, the MR-Egger method assumes complete invalidity of these variants, providing a robust alternative for assessing causality amidst potential pleiotropy. To minimize false-positive results, associations with multiple Bonferroni correction thresholds (*P* < 0.05/1,400) were considered significant, while associations with *P* < 0.05 were considered suggestive.

### 2.5 Sensitivity analysis

Sensitivity analysis was used to further evaluate any deviations from the MR hypothesis. Cochran’s Q test was used to evaluate the heterogeneity among the IVs. The non-zero intercept observed in the MR-Egger regression analysis suggests the potential for horizontal pleiotropy, a phenomenon where a SNP exerts influence over multiple distinct phenotypes. MR pleiotropy residual sum and outlier analysis (MR-PRESSO) provides causal estimates after removing outliers. The Steiger test is employed to assess the directionality of the impact of IVs on the outcomes and to reject the resulting bias caused by reverse causality. The leave-one-out method was used to identify IVs that deviated significantly from the others.

### 2.6 Post-Mendelian randomization meta-analysis

To validate the significant associations identified in the primary analysis, we used additional independent AD GWAS data from the FinnGen Consortium to replicate the IVW analysis. Subsequently, a meta-analysis was conducted to identify the final candidate blood metabolites.

### 2.7 Reverse Mendelian randomization analysis

To determine whether previously identified significant causal relationships exhibit reverse causal interference that violates the MR hypothesis, we conducted a reverse MR analysis using AD as the exposure and examined its causal effects on metabolites. SNPs that showed a significant association with AD across the entire genome were chosen (*P* < 5 × 10^–8^).

### 2.8 Multivariable Mendelian randomization analysis

To investigate the direct and autonomous causal connection between candidate metabolites and AD, the primary analytical method utilized was MVMR-IVW, which was complemented by the MVMR-Egger, MVMR-Lasso, and MVMR-median methods for validation.

## 3 Results

### 3.1 Screening of instrumental variables

After IV screening, 4-56 SNPs strongly related to blood metabolites and metabolite ratios were also identified. For reverse MR analysis, we identified 62 SNPs strongly associated with AD. The F-statistics for all IVs were greater than 10, indicating that weak instrumental variable bias was excluded. The harmonized data are presented in Supplementary Tables 2A, B.

### 3.2 Primary analysis and sensitivity analysis

**FIGURE 2:**
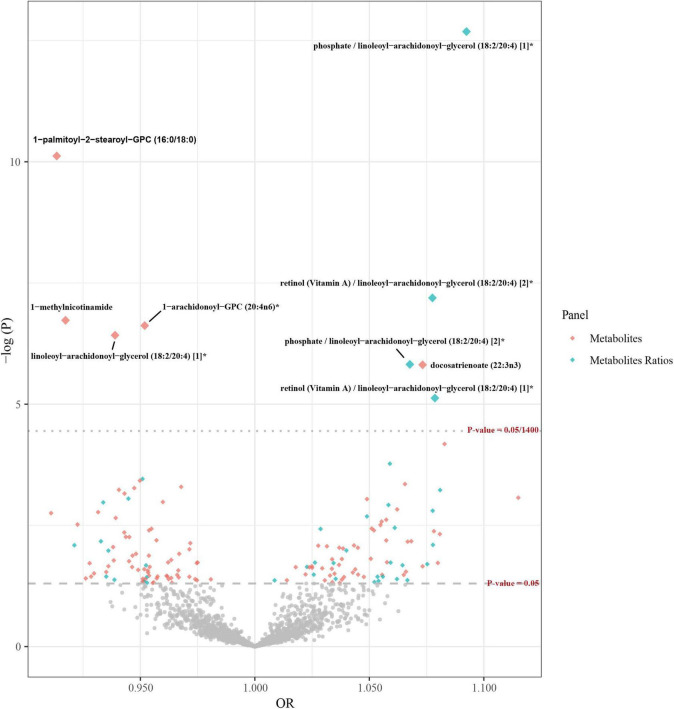
Volcano plots of Mendelian randomization in the primary analysis. The *x*-axis represents the odds ratio, and the *y*-axis represents the negative logarithm of the *P*-value. The gray rhombic dots indicate that their *p*-values are below the threshold of 0.05, while the colorful large dots indicate that their *p*-values are above the threshold of 0.05/1,400, which is the threshold after multiple corrections using the Bonferroni method. The colorful small dots indicate that their *p*-values are between the thresholds of 0.05 and 0.05/1,400.

In the preliminary analysis, the IVW method was used as the main analysis method, while MR-Egger and WM were employed as supplementary methods. Multiple test correction was performed using the Bonferroni method (as shown in Figure 2), and a set of sensitivity analyses was carried out. Partial results meeting the following criteria were excluded: (1) Results showing inconsistent directions of causal relationship estimates identified by the three analytical methods (Supplementary Figure 1). (2) If the Egger intercept *P* is less than 0.05 or the MR-PRESSO global test *P* is less than 0.05, horizontal pleiotropy is indicated. (3) The Steiger test indicates that the causal direction is false. (4) Results identified by the leave-one-out method with outliers. In the forward MR analysis investigating the causal relationship between blood metabolites and AD, the IVW method revealed five blood metabolites and metabolite ratios significantly associated with an increased risk of AD: docosatrienoate (22:3n3) (odds ratio (OR) = 1.07, 95% confidence interval (CI) 1.04–1.11, *P* = 1.54 × 10^–6^), phosphate/linoleoyl-arachidonoyl-glycerol (18:2/20:4) (OR = 1.09, 95% CI 1.07–1.12, *P* = 2.06 × 10^–13^), retinol (VA)/linoleoyl-arachidonoyl-glycerol (18:2/20:4) [2]* (OR = 1.08, 95% CI 1.05–1.11, *P* = 6.42 × 10^–8^), phosphate/linoleoyl-arachidonoyl-glycerol (18:2/20:4) (OR = 1.07, 95% CI 1.04–1.10, *P* = 1.51 × 10^–6^), and retinol (Vitamin A)/linoleoyl-arachid At the same time, four blood metabolites and metabolite ratios that are significantly related to reducing the risk of AD were identified. Specifically, 1-palmitoyl-2-stearoyl-GPC (16:0/18:0) (OR = 0.91, 95% CI 0.89–0.94, *P* = 7.59 × 10^–11^), 1-methylnicotinamide (OR = 0.92, 95% CI 0.89–0.95, *P* = 1.86 × 10^–7^), 1-arachidonoyl-GPC (20:4n6)* (OR = 0.95, 95% CI 0.93–0.97, *P* = 2.39 × 10^–7^), and linoleoyl-arachidonoyl-glycerol (18:2/20:4) [1]* (OR = 0.94, 95% CI 0.92–0.96, *P* = 3.78 × 10^–7^) were used. Furthermore, 148 potential causal associations were identified (as shown in Supplementary Tables 3A, B). However, in leave-one-out analyses, we observed that rs174566 significantly influenced the causal inference of the lipid species 1-palmitoyl-2-arachidonoyl-GPC (16:0/20:4n6). Similarly, rs73154060 was found to exert a significant impact on the metabolite ratio of pyruvate to 3-methyl-2-oxobutyrate. Additionally, rs174564 was identified as a significant determinant of the causal inference of the arachidonate (20:4n6) to linoleate (18:2n6) ratio. Given the potential for these SNPs to compromise the robustness of our conclusions, the associated phenotypes were deliberately excluded from further analysis (Supplementary Figure 2).

### 3.3 Post-Mendelian randomization meta-analysis

**FIGURE 3:**
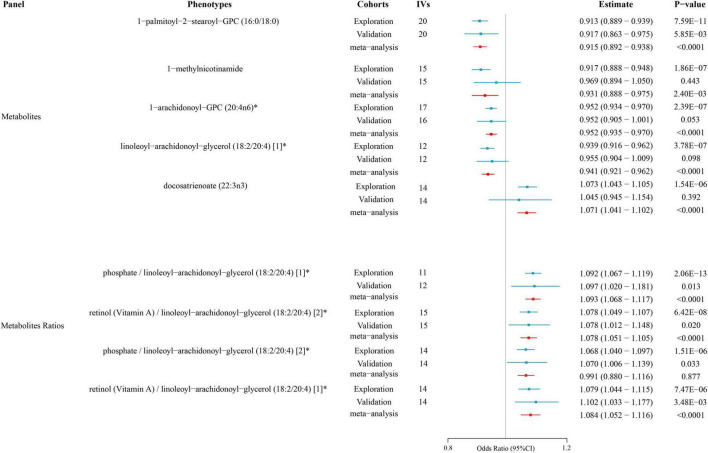
Causal relationships between metabolites and atopic dermatitis according to Mendelian randomization. The effect estimates (Estimate) and corresponding *P*-values for various metabolites and metabolite ratios are shown. The results from the exploration cohort, validation cohort, and meta-analysis are depicted to assess the consistency across different cohorts.

To enhance the credibility of the findings from the primary analysis, we included additional independent AD GWAS data from the FinnGen consortium for meta-analysis. The results pointed to the same direction as the preliminary analysis’s estimate of cause and effect. There were also statistically significant results (*P* < 0.05) for all metabolites. These results still pointed in the same direction as the preliminary analysis’s estimates of cause and effect. Therefore, we can include them as potential metabolites for further analysis (as shown in Supplementary Tables 3C, D) (Figure 3).

### 3.4 Reverse Mendelian randomization analysis

Our results from the reverse MR analysis, outlined in Supplementary Table 4A, demonstrated that phosphate/linoleoyl-arachidonoyl-glycerol (18:2/20:4) [1]*, linoleoyl-arachidonoyl-glycerol (18:2/20:4) [1]*, and retinol (vitamin A)/linoleoyl-arachidonoyl-glycerol (18:2/20:4) [1]* were associated with AD. However, findings from the Steiger test indicated that IVs accounted for a lesser degree of variation in AD than metabolites. This discrepancy suggests that the presumed causal direction may be erroneous, thereby undermining the robustness of reverse MR causal inference.

### 3.5 Multivariable Mendelian randomization analysis

After MVMR correction of five candidate metabolites, the IVW method revealed that the levels of 1-methylnicotinamide (OR = 0.92, 95% CI 0.88–0.96, *P* = 7.93 × 10^–4^) and 1-palmitoyl-2-stearoyl-GPC (16:0/18:0) (OR = 0.94, 95% CI 0.90–0.99, *P* = 9.98 × 10^–3^) were protective against AD on their own. The consistency in the direction of the MVMR-Egger, MVMR-Lasso, and MVMR-median effect estimates further supports our conclusions. However, after adjusting for multiple variables for the ratios of the four candidate metabolites, we found no independent causal effect (as shown in Supplementary Tables 5A, B).

## 4 Discussion

The main objective of this research was to offer valuable insights and strategies to improve the diagnosis and management of AD. Dysregulation of blood metabolites can result in immune-related disorders. Due to its potent anti-inflammatory properties, nicotinamide (NA) has been proposed as a potential treatment for AD. Research has indicated that increased levels of niacinamide and its derivatives in mothers are linked to a decreased likelihood of AD in 12-month-old infants (10). At the same time, topical NA application simultaneously maintains the skin barrier, decreases transepidermal water loss (TEWL), and enhances skin protein and ceramide synthesis. This, in turn, enhances the resistance of the skin to environmental allergens and pathogens, alleviating symptoms of AD (11, 12). 1-Methyl nicotinic acid (1-MNA) has also been shown to have significant anti-inflammatory effects in AD due to its chemical stability, nontoxicity, and well-tolerated nature (13–16). This finding supports our conclusion that elevated levels of 1-MNA lead to a reduced risk of AD development. Notably, unlike its precursor NA, 1-MNA does not directly inhibit immune cell functions, such as the production of proinflammatory cytokines and mediators (17). In contrast, its anti-inflammatory properties might be connected to its impact on the endothelial cells lining blood vessels (18). Our hypothesis is that 1-MNA has the potential to function as a scavenger by removing reactive oxygen species such as superoxide radicals, hydroxyl radicals, and anions from proinflammatory molecules on the surface of vascular endothelial cells. This suggests that 1-MNA exerts powerful anti-inflammatory effects while minimizing damage to the body’s immune system. The potent anti-inflammatory and antioxidant properties of 1-MNA, along with its potential to enhance skin barrier function, make it a promising topical agent for treating AD.

In addition, it is worth noting that most metabolites or rates of metabolites that reduce the risk of developing AD include arachidonic acid (AA) or its derivatives. For example, we found that elevated levels of linoleoyl-arachidonoyl-glycerol (18:2/20:4) were linked to a reduced risk of developing AD. This may be due to the presence of diacylglycerol in edible vegetable oils. The consumption of diacylglycerol has been proven to enhance metabolic syndrome by decreasing postprandial hyperlipidemia and hemoglobin A1C levels, boosting energy expenditure, and reducing obesity caused by food. Interestingly, a large number of observational studies have shown an association between overweight or obesity and AD, with the association being stronger in women than in men (19, 20). In addition, MetS was positively associated with the presence of AD in women, even after adjusting for confounders (21, 22). AD is a source of activity for a variety of proinflammatory cytokines and chemokines and is typically mediated by Th2 cells, leading to elevated interleukin-4 and IL-13 concentrations. It is often comorbid with Mets (23, 24). The generation of proinflammatory cytokines is triggered in adipose tissue through the release of soluble adipokines, which play a role in immunity and inflammation. This may lead to persistent, mild inflammatory conditions and heightened vulnerability to allergic reactions. Diacylglycerol, on the other hand, indirectly reduces the risk of AD by decreasing systemic inflammation and oxidative stress linked to obesity and metabolic disorders. In addition, we note that linoleoyl-arachidonoyl-glycerol (18:2/20:4) contains linoleic acid (LA) molecules in its internal structure. LA constitutes the highest concentration of fatty acid in the epidermis, with its various compounds serving as crucial components in both the composition and operation of the epidermal barrier. Water-in-oil emulsions containing LA have been shown to reduce erythema (24). This suggests that unsaturated fatty acids, such as LA, may improve the skin condition and regulate the immune system in patients with AD. Therefore, elevated levels of linoleoyl-arachidonoyl-glycerol (18:2/20:4) [1]* may reduce the risk of AD incidence. AD may be treated by targeting the inflammatory pathway shared by AD patients and MetS patients, as well as by moderately supplementing patients with LA in the future.

In addition, the internal structure of phosphate/linoleoyl-arachidonoyl-glycerol (18:2/20:4) [1]* contains arachidonic acid esters (20:4n6) and LA esters (18:2n6), both of which are omega-6 polyunsaturated fatty acids (n-6 PUFAs). A rigorous RCT revealed long-term symptom improvement in some patients with AD after intravenous administration of n-6 PUFAs (25). Furthermore, the epidermal phosphoglycerides of patients with AD exhibited a significant increase in monounsaturated fatty acids and a significant decrease in n-6 fatty acids compared to those of lesion-free epidermis (26). The contribution of n-3 and n-6 PUFAs to the proper structure, elasticity, and function of cell membranes could be the reason behind this phenomenon. These fatty acids play a crucial role in intracellular lipid synthesis within the epithelial stratum corneum, aiding in the maintenance of the permeability barrier of the stratum corneum and ultimately decreasing the prevalence of AD. GLA is an n-6 PUFA with anti-inflammatory properties that increases ceramide synthesis (27). A decrease in ceramide levels is a crucial factor in the emergence of abnormal skin conditions, such as dryness and increased TEWL, in individuals with AD skin (28, 29). This finding supports our conclusion that n-6 PUFAs are protective factors for AD. Notably, our study revealed that an increase in the ratio of arachidonate (20:4n6)/linoleate (18:2n6) among the n-6 PUFAs reduced the risk of AD development. Previous studies on eczema have shown that LA intake is positively associated with the development of eczema, while arachidonic acid intake reduces the risk of its development (30). Although AA can generate arachidonoids, such as prostaglandins (PGs), thromboxanes, and leukotrienes, via the COX pathway, these molecules serve as key mediators of allergic inflammation and are generally believed to promote chronic inflammation in AD. On the other hand, n-3 PUFAs exert anti-inflammatory effects by inhibiting AA incorporation into cell membranes and AA metabolism into arachidonoids (31, 32). However, it should be noted that certain PGs, such as PGE2 and PGI2, inhibit inflammatory responses induced by allergens (30). Lipoxin A4 is an important bioactive molecule generated by AA through the action of lipoxygenase. Lipoxin A4, as opposed to LTs, exerts its anti-inflammatory effects by inhibiting the synthesis of chemokines and inflammatory cytokines. Therefore, elevated levels of arachidonate (20:4n6) may alleviate chronic inflammation in AD through the generation of PGs with anti-inflammatory effects, as well as through the lipoxygenase pathway.

Our approach to translating the current findings into clinical practice is structured around a comprehensive translational research strategy, encompassing preclinical validation through to phase IV clinical trials, with an emphasis on precision medicine. The roadmap outlined below aims to guide subsequent researchers in translating our current findings into tangible clinical advancements, traversing the stages of preclinical validation through to phase IV clinical trials, with a strong emphasis on precision medicine strategies. Given the ethical considerations inherent in clinical research, it is imperative that future studies first conduct a rigorous preclinical assessment of the efficacy and safety of 1-MNA and other promising metabolites in relevant AD animal models. This foundational step is essential for establishing the scientific basis upon which human trials can be justified. Building on this preclinical foundation, phase I clinical trials should focus on the safety, tolerability, and initial pharmacokinetics of these metabolites in both healthy volunteers and early-stage AD patients. Subsequently, phase II trials should be designed to evaluate the efficacy of these metabolites in a larger cohort of AD patients, employing stringent outcome measurements such as the SCORAD index, TEWL, and skin hydration levels to assess clinical efficacy accurately. Progressing further, phase III trials should emphasize large-scale, multicenter studies, including cohort studies or randomized controlled trials, to verify the therapeutic benefits across diverse populations, thereby providing the necessary evidence for regulatory approval. Following approval, phase IV post-marketing surveillance is crucial for monitoring the long-term sustainability of treatment effects, identifying any late-emerging adverse reactions, and evaluating the overall impact on the quality of life of AD patients. Most importantly, acknowledging the genetic and metabolic heterogeneity of AD, future trials should explore patient stratification strategies based on individual metabolic profiles and genetic susceptibilities. This approach is aimed at achieving precision medicine for AD patients, with the ultimate goal of improving cure rates and prognosis.

We included the largest collection of nontargeted plasma metabolome data and the most recent and most comprehensive AD-GWAS data to date, overcoming the inherent shortcomings of traditional observational studies. To ensure the robustness and reliability of the results, multiple test calibration and sensitivity analysis methods were used. Nevertheless, it is crucial to acknowledge that this study was subject to specific limitations. Firstly, to ensure the inclusion of an adequate number of IVs for analysis, a lenient significance level (*p* < 5 × 10^–6^) was used. Secondly, while MR is based on the genetic principle that “allelic genes are randomly distributed to offspring during gamete formation,” thereby minimizing the influence of environmental and socioeconomic confounders, our study may still be subject to unidentified biases due to the absence of individual-level data that could account for all potential confounding factors. Future studies should endeavor to incorporate such data to enhance the precision of causal inferences. Thirdly, Given that the publicly available GWAS databases from which we sourced our data are predominantly composed of individuals of European descent, our study may be limited in its ability to extrapolate findings to other ethnic populations. The disparity in socioeconomic status across different ethnicities can lead to variations in access to healthcare, health awareness, and disease management practices, potentially impacting disease incidence rates. Furthermore, the criteria for disease definition and diagnosis may vary by region and ethnicity, affecting the identification and classification of conditions within different populations. Most critically, due to gene-environment interactions, certain genetic variants associated with diseases or metabolites may exhibit different distributions across ethnicities, which could alter the inference of causal relationships. Therefore, it is imperative that future research includes assessments of ethnic differences to validate the broader applicability of our findings. Fourthly, further clinical and pharmaco epidemiologic studies, as well as large-scale RCTs, could be used to validate our conclusions in the future.

## 5 Conclusion

In this study, significant causal associations between genetically related blood metabolites and AD at the genetic and metabolic levels were identified through bidirectional MR and MVMR analysis in European population, and a meta-analysis was used to confirm the findings of the preliminary investigations. These results provide important insights into dietary modifications and nutritional interventions for AD patients in Europe and potential biological markers for the development of metabolomic-based therapies. Further research is necessary to validate these findings in different populations and clarify the mechanisms underlying the predicted link between blood metabolites and AD.

## Data Availability

The original contributions presented in this study are included in this article/Supplementary material, further inquiries can be directed to the corresponding author.

## Supplementary material

Supplementary Figure 1 Scatter plots of metabolite levels in atopic dermatitis patients according to the primary analysis. Each subplot **(a-i)** corresponds to a different metabolite or metabolite ratio. The *x*-axis represents the effect size of the single nucleotide polymorphism (SNP) on the respective metabolite, while the *y*-axis indicates the effect size of the SNP on the AD. The slope of the line represents the causal association, and each method has a distinct line. The consistency of the five methods proves that the results are stable and reliable.

Supplementary Figure 2 Leave-one-out analyses of metabolite levels in atopic dermatitis patients in the primary analysis. Each subplot **(a-i)** represents various metabolite ratios or metabolites, showing the effect size change when each SNP was omitted from the analysis. The lines or points indicate the correlation strength between the remaining SNPs and the metabolite ratios or AD after the exclusion of a particular SNP.
